# Autopsy Report of a 7-Year Old Patient with the Mosaic Trisomy 13

**DOI:** 10.1007/s12013-013-9567-y

**Published:** 2013-03-24

**Authors:** George Imataka, Hideo Yamanouchi, Junko Hirato, Mitsuoki Eguchi, Masaru Kojima, Koichi Honma, Osamu Arisaka

**Affiliations:** 1 Department of Pediatrics, Dokkyo Medical University School of Medicine, 880 Mibu, Shimotsuga, Tochigi, 321-0293 Japan; 2 Department of Pediatrics, Saitama Medical University, Saitama, Japan; 3 Department of Human Pathology, Gunma University Graduate School of Medicine, Gunma, Japan; 4 Department of Pediatrics, International University of Health and Welfare Shioya Hospital, Tochigi, Japan; 5 Department of Anatomic and Diagnostic Pathology, Dokkyo Medical University School of Medicine, Tochigi, Japan

**Keywords:** Trisomy 13, Patau syndrome, Survival, Autopsy, Olfactory aplasia, Torpedos

## Abstract

We present here a long survival case of a patient with the mosaic form of trisomy 13 who died of aspiration pneumonia at the age of 7 years and 4 months. The autopsy revealed olfactory aplasia and fenestration of the septum pellucidum, and dilated lateral ventricles and atrophic hippocampus. Furthermore, there were numerous “torpedos” (i.e., swollen fusiform Purkinje cell axons), mostly in the granular layer underneath the Purkinje cell layer, and, occasionally, in the granular layer. Similar neuropathological findings have been reported in elderly cases of essential tremor, Parkinson’s disease, or Alzheimer’s disease. Precise mechanism for this axonal change is still unclear. These pathological changes have never previously been reported in the literature on trisomy 13, and the present patient is one of the oldest autopsied individuals with the mosaic trisomy 13.

## Introduction

The trisomy 13 syndrome, also called Patau syndrome, was first reported in 1960 [1]. It is a congenital anomaly syndrome caused by accessory chromosome 13 of the D1 group. Clinically, this syndrome is manifested by variable deformities and their complications. Most patients with this syndrome die soon after birth because of the severe congenital heart disease and brain deformity. The mortality rate is approx. 50 % in the first month of life and 90 % during the first year of life [2]. Although a few cases of longer survival have been reported [3, 4], there have been no published reports to date on neuropathological findings in long survival cases with trisomy 13. Here, we report an autopsy case of a 7-year-old boy with mosaic type of the trisomy 13 syndrome. This autopsy in one of the longest survival cases in the literature.

## Patient’s History

**Fig. 1:**
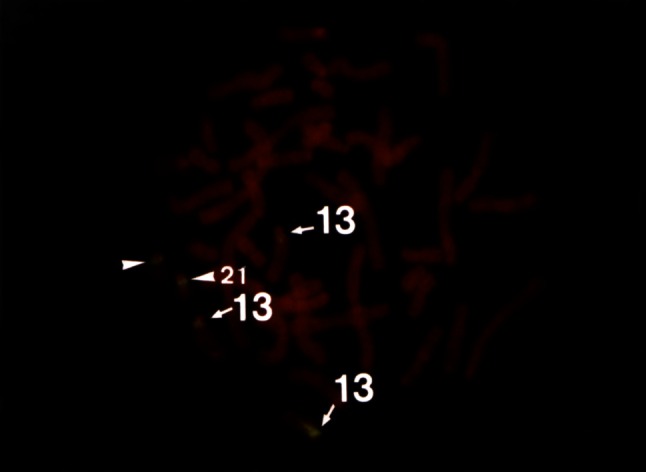
Fluorescence in situ hybridization (FISH) reveals mosaic trisomy 13, with 73.2 % of cells carrying normal karyotype and 26.8 % trisomy 13

This patient was born vaginally after 39 weeks of gestation weighting 3,366 g. He was immediately transported to our Hospital because of neonatal aspiration and meconium aspiration syndrome. The boy had systolic heart murmurs and was further diagnosed with ventricular septal defect type IV. He also had several minor anomalies, including capillary hemangioma on forehead, frontal alopecia, narrow V-shaped palate, narrow fingernails, cryptorchidism, inguinal hernia, and penis palmatus. Cytogenetic analysis was carried out using a peripheral blood specimen, and the G-banding karyotype analysis revealed the presence of trisomy 13. We did not conduct the chromosome analysis of buccal mucosa or skin fibroblasts. Fluorescence in situ hybridization (FISH) assay showed mosaic trisomy 13: 73.2 % of cultured cells had the karyotype 13 (47, XY + 13), while the remainder of the cells had normal karyotype (Fig. 1). Convulsions developed in the infancy. Electroencephalogram showed multifocal spikes; the convulsions were well controlled by 0.05 mg/kg/day of clonazepam. At the age of 3 years, repeated respiratory infections were seen, so that the boy frequently needed mechanical respiratory support. Respiratory problems were eventually controlled by tracheotomy, following which the patient was able to stay at home. At the age of 5 years, there were frequent urinary infections due to penis palmatus. These infections ceased after urethroplasty. At the age of 7 years, the patient could roll over his body and enjoyed touching the toys. Physical examination revealed poorly developed and underweight child with respiratory failure. Severe aspiration pneumonia developed at the age of 7 years and 4 months, leading to the patient’s demise.

## Neurological Evaluation at the Age of 7 Years

**Fig. 2:**
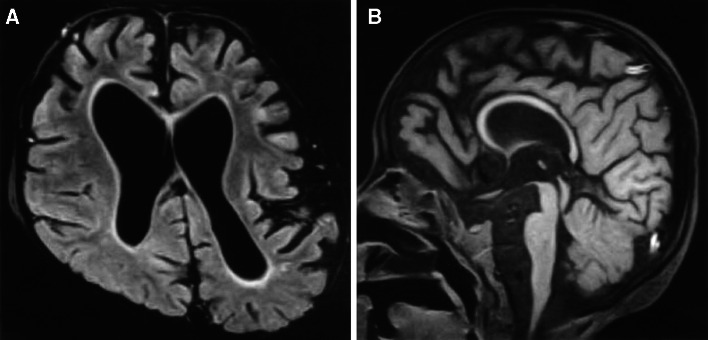
**a** Magnetic resonance imaging (MRI), T_1_-weighted, 1.5T axial view (*SE* spin echo; TR = 15 ms; TE = 450 ms). MRI reveals dilatations of lateral ventricles and bifurcated septum pellucid. **b** The splenium of the corpus callosum is thin

**Fig. 3:**
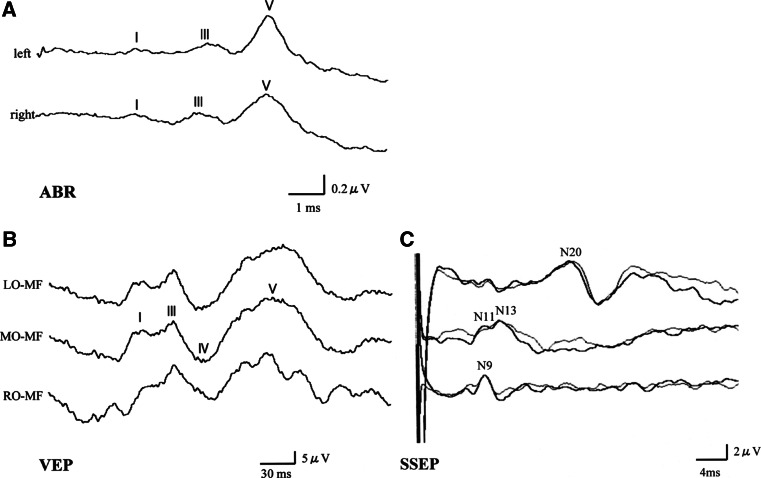
**a** The auditory brainstem response to 100 dB reveals prolonged wave I latency (2.9 and 3.0 ms in the *left* and *right* sides, respectively). **b** The visual-evoked potential shows intact wave components with delayed latency. **c** The short-latency somatosensory evoked potential demonstrates prolonged central conduction time (7.04 ms)

From the age of 6 years, the patient developed aggravated epileptic seizures accompanied by partial convulsions of the left arm. Repeated electroencephalogram (EEG) spikes in the centroparietal area of the right hemisphere were observed. Followed the diagnosis of focal epilepsy, the dose of clonazepam was increased up to 0.1 mg/kg/day which led to cessation of seizures. At the age of 7 years, the drug-induced sleep EEG showed no spikes. On the extremities’ examination, spastic paralysis of lower extremities and increased deep-tendon reflex were observed. Furthermore, neurological examination showed brisk deep-tendon reflexes and a rigid and spastic muscle tone. The examination of the cranial nervous system revealed declined activities of the facial expression muscles, diminished eyelash reflex, and attenuated vomiting reflex. The pupil dilation response was slow bilaterally. The ophthalmoscopy revealed atrophic changes in bilateral optic nerves. The brain MRI showed no brain malformation (Fig. 2a, b), and the brain showed relatively good myelination. Both left and right lateral ventricles were enlarged, and there were hypoplasia of transparent septum and thinning of the splenium of the corpus callosum. The auditory brainstem response to 100 dB revealed prolongation of bilateral I wave latency at 2.9 and 3.0 ms for the left and right side, respectively (Fig. 3a). However, the central conduction time and the I–V wave latencies were normal, with no difference between the left and right side of the boy’s brain (3.8 and 3.7 ms, respectively). The amplitudes of the II, III, and IV waves were low. Components of the normal reaction wave were seen on the visual evoked potential (Fig. 3b) and electroretinogram. Prolongation was observed in the central conduction time at 7.04 ms (+2.8 SD) upon short-latency somatosensory evoked potential (Fig. 3c).

## Blood Cell Morphology at the Age of 7 Years

**Fig. 4:**
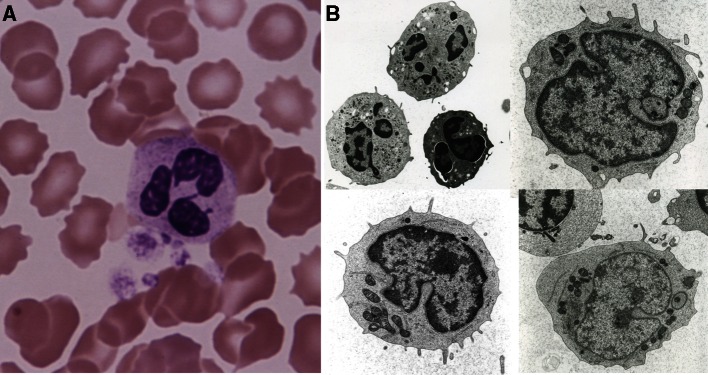
**a** Hematoxylin–eosin staining of peripheral blood neutrophils reveals hypersegmentation and drumsticks of neutrophil nuclei (magnification ×100). **b** Electron microscopy (JOEL, Tokyo, Japan; magnification ×100) reveals nuclear pockets and dendritic deformation of mitochondria

Nuclear hypersegmentation and drumstick deformation with club-shaped protrusions were frequently observed in peripheral blood neutrophils (Fig. 4a). Blood cells were fixed in 1 % osmium tetroxide, dehydrated with ethanol, and embedded in epoxy resin. Then, ultrathin (100 nm) sections were prepared and double-stained with uranyl acetate and lead citrate. These specimens were observed using electron microscopy, which revealed small protrusions of neutrophil nuclei, nuclear pockets, and dendritic deformation of the mitochondria (Fig. 4b).

## Neuropathology

**Fig. 5:**
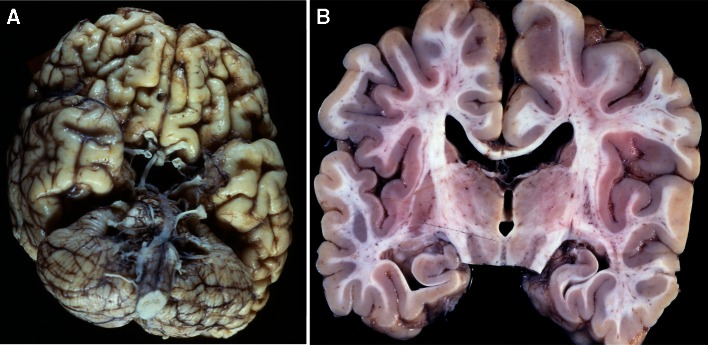
**a** The olfactory bulb, tract, trigone, and anterior perforated substance are absent, and gyrus rectus is abnormally shaped. **b** Coronal section of the autopsied brain demonstrates fenestration of the septum pellucidum

Autopsy was performed soon after death. The boy was 93 cm tall and weighed 6 kg. The brain weight was not measured. Cerebral hemisphere showed symmetric atrophy with widened sulci and narrow gyri. The olfactory bulb, tract, and trigone, and anterior perforated substance were absent, and gyrus rectus showed shape deformation (Fig. 5a). The septum pellucidum was fenestrated, and corpus callosum was thin (Fig. 5b).

**Fig. 6:**
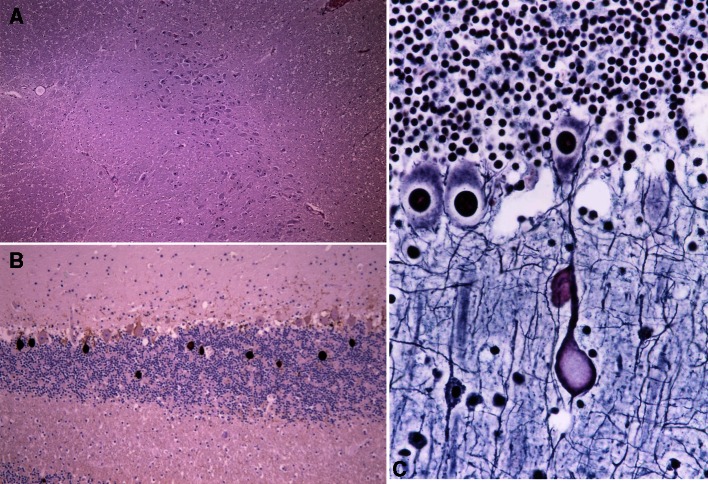
**a** Decreased melanin granule is observed in pars compacta of the substantia nigra (hematoxylin–eosin staining; ×100). **b** The presence of characteristic axonal swelling of Purkinje cells (torpedoes) (neurofilament; ×100). **c** Similar axonal swelling of Purkinje cells is seen in the molecular layer (Bodian; ×200)

Histopathologically, each layer of the cerebral cortex was preserved, and polarity of pyramidal cells was intact. The melanin granule was absent in pars compacta of the substantia nigra (Fig. 6a). A large number of torpedoes (swollen axons of Purkinje cells) were seen in the granular layer below the Purkinje cell layer (Fig. 6b). Similar axonal swelling was seen even in the molecular layer, albeit not as extensive (Fig. 6c). There were numerous spheroids in the gray matter of the spinal cord and medulla.

## Discussion

A commonly encountered brain anomaly in trisomy 13 is holoprosencephaly, which is a brain disorder characterized by a failure of differentiation and a various degree of cleavage of the prosencephalon [5]. This patient exhibited olfactory aplasia and partial dysgenesis of the septum pellucidum. However, there was no fusion of cerebral hemispheres nor a partial or complete absence of the corpus callosum. Olfactory aplasia is sometimes associated with septo-optic dysplasia [6]. However, this diagnosis was not applicable to our patient because of incomplete absence of septum pellucidum. Furthermore, the autopsy revealed a relatively well-preserved optic tract. Isolated olfactory aplasia has never previously been reported in trisomy 13.

The mortality is generally associated with the severity of deformities in the central nervous system, heart, and respiratory system. To the best of our knowledge, our patient is one of the longest survivors with trisomy 13 subjected to autopsy. The patient’s long survival was due to the absence of complications associated with deformities of major organs, such as the brain, the heart, or the lungs. Another unique feature in our case was the presence of numerous torpedoes, i.e., swollen axons of Purkinje cells, within the granular layer. The precise mechanism for this axonal change remains unknown. It has been reported in several neuropathologic conditions with diffuse or focal cerebellar changes, such as necrotic lesions, degenerative disease involving the cerebellum, elderly cases of essential tremor, Parkinson’s disease, Alzheimer’s disease, cerebellar injury, and aging [7–9]. However, this characteristic change in the cerebellum has never previously been reported in autopsy reports on trisomy 13.
